# Association between frailty and cognitive impairment in elderly patients with acute cerebral infarction

**DOI:** 10.3389/fnagi.2026.1709355

**Published:** 2026-02-20

**Authors:** Xiangjun Tao, Ying Wang, Shu Ding

**Affiliations:** 1 Department of Nursing, Beijing Chao-Yang Hospital, Capital Medical University, Beijing, China; 2 Department of Neurology, Beijing Chao-Yang Hospital, Capital Medical University, Beijing, China

**Keywords:** acute cerebral infarction, cognitive impairment, Edmonton Frail Scale, elderly, frailty, Montreal Cognitive Assessment

## Abstract

**Background:**

Post-stroke cognitive impairment is common in older patients, yet the interaction between frailty and cognition remains insufficiently characterized. Evidence using multidimensional frailty tools during hospitalization for acute cerebral infarction is limited, and domain-level cognitive correlates of frailty have not been well described.

**Methods:**

In this hospital-based cross-sectional study, consecutive patients aged ≥65 years with acute cerebral infarction were enrolled. Frailty (Edmonton Frail Scale, EFS) and cognition (Montreal Cognitive Assessment, MoCA) were assessed 7–14 days after admission. Cognitive impairment (CI) was defined as MoCA <26, with a 1-point adjustment for ≤12 years of education applied before group assignment. Associations between EFS total score and MoCA domains were evaluated using Spearman correlations. Multivariable logistic regression was used to identify independent predictors of CI, with alcohol intake modeled per 10 g ethanol/day.

**Results:**

Among 179 participants, 117 (65.4%) had CI and 62 (34.6%) were cognitively unimpaired (CU). Patients with CI were older than CU patients (73.0 ± 5.9 vs. 71.3 ± 4.2 years) and had higher admission NIHSS scores (3.85 ± 2.80 vs. 2.77 ± 2.61). The prevalence of pre-frailty/frailty (EFS ≥ 6) was 45.8% overall and was higher in the CI group than in the CU group (59.8% vs. 19.4%). EFS total score was inversely correlated with global cognition and all MoCA domains (total MoCA: *ρ* = −0.572, *p* < 0.001). In multivariable analysis (*n* = 173 complete cases), higher alcohol intake (per 10 g ethanol/day; OR 1.43, 95% CI 1.07–1.90) and higher frailty score (OR 1.34, 95% CI 1.17–1.55) were independently associated with CI, whereas higher education level was protective (OR 0.59, 95% CI 0.37–0.94).

**Conclusion:**

In older patients hospitalized with acute cerebral infarction, cognitive impairment showed an association with higher frailty burden and higher alcohol intake, supporting integrated frailty and alcohol exposure assessment during early in-hospital evaluation.

## 1 Introduction

Acute cerebral infarction (ACI) is a leading cause of mortality and long-term disability worldwide, and following ACI, many patients experience reduced quality of life (Costa Novo et al., 2024; Xu et al., 2024). The burden of this condition is amplified in older adults, consistent with the rising prevalence of age-related conditions globally (Luo et al., 2021; Guo et al., 2023). Beyond motor and functional deficits, cognitive impairment is a frequent and clinically important complication after infarction, adversely affecting rehabilitation participation, medication adherence, independence in activities of daily living, and quality of life (Guo et al., 2023). Early identification of patients at higher risk for post-stroke cognitive impairment may facilitate timely cognitive rehabilitation and individualized secondary prevention strategies.

Reported estimates of post-stroke cognitive impairment vary widely across studies. At approximately 3 months after stroke, prevalence has been reported to range from about 17% to over 92% (Brainin et al., 2015), a wide range likely driven by differences in case definitions, assessment tools, and timing of evaluation. This heterogeneity complicates clinical risk stratification and underscores the need for studies that apply standardized cognitive screening approaches and clearly define the assessment window, particularly in older inpatient cohorts.

Frailty is a multidimensional geriatric syndrome characterized by diminished physiological reserve and increased vulnerability to stressors. Frailty is distinct from multimorbidity and disability, but often coexists with both (Singh et al., 2014). In older adults, frailty has been linked to adverse outcomes including falls, hospitalization, mortality, and impaired recovery following acute illness (Burton et al., 2022). In the context of stroke, frailty may capture pre-morbid vulnerability and post-event deconditioning, potentially shaping neurocognitive resilience and recovery—a link suggested by evidence associating frailty with incident cognitive disorders and dementia progression in other cohorts (Taylor-Rowan et al., 2019, 2023). However, the interaction between frailty and cognition in acute infarction remains insufficiently characterized. Specifically, evidence using multidimensional frailty tools during hospitalization for ACI is limited, and few studies have examined how frailty relates to domain-level cognitive performance rather than only global cognitive scores.

The Montreal Cognitive Assessment (MoCA) is widely used for post-stroke cognitive screening and enables evaluation across multiple cognitive domains, while the Edmonton Frail Scale (EFS) provides a brief multidimensional assessment of frailty suitable for clinical settings. Integrating these tools may offer a pragmatic approach to identify older stroke patients who may benefit from early geriatric-informed interventions. In parallel, lifestyle factors such as alcohol intake may contribute to cognitive vulnerability, yet alcohol exposure is often reported with unclear units or clinically uninterpretable increments, limiting translation of findings into practice.

Therefore, we aimed to (1) quantify the prevalence of cognitive impairment and pre-frailty/frailty in older patients hospitalized with acute cerebral infarction, (2) examine the association between multidimensional frailty (EFS) and domain-level cognitive performance (MoCA), and (3) identify factors independently associated with cognitive impairment, with alcohol intake expressed in clinically interpretable units (grams of ethanol per day).

## 2 Materials and methods

### 2.1 Study design

This hospital-based, cross-sectional observational study was conducted at the Department of Neurology, Beijing Chao-Yang Hospital, from February 2018 to June 2023. The study protocol was approved by the Institutional Ethics Committee of Beijing Chao-Yang Hospital (Approval No. 2025-KE-567). Written informed consent was obtained from all participants or their legally authorized representatives.

### 2.2 Participants

Consecutive patients aged 65 years or older who were admitted with acute cerebral infarction (ACI) were screened for eligibility. ACI was diagnosed based on neurological examination and brain imaging (CT and/or MRI) in accordance with World Health Organization criteria.

Inclusion criteria were: (1) age ≥ 65 years; (2) imaging-confirmed ACI on admission; (3) ability to complete cognitive and frailty assessments during hospitalization; and (4) provision of written informed consent.

Exclusion criteria were: (1) history of intracranial hemorrhage, transient ischemic attack, or major neurological disorders (e.g., Parkinson disease, prior dementia, or severe traumatic brain injury); (2) severe aphasia, dysarthria, or visual/hearing impairment precluding valid neuropsychological testing; (3) severe psychiatric illness (e.g., schizophrenia or major depressive disorder); (4) acute severe infection, systemic inflammatory disease, or major organ dysfunction at admission; (5) active malignancy, hematologic disease, or use of immunosuppressive agents or systemic corticosteroids within the preceding 3 months; and (6) refusal or inability to provide informed consent. Prior dementia and major depression were screened by medical record review and caregiver history. Acute severe infection and major organ dysfunction were determined by attending physician diagnosis and admission laboratory findings.

All cognitive and frailty assessments were performed 7–14 days after admission to allow for neurological stabilization. This assessment window may have excluded the most severe cases; the number of patients unable to complete testing was documented.

### 2.3 Clinical and demographic data

Demographic characteristics (age, sex, body mass index, and education level), lifestyle factors (smoking and alcohol intake), vascular risk factors and comorbidities, medication history, and stroke severity assessed by the National Institutes of Health Stroke Scale (NIHSS) on admission were recorded. Infarction sites were determined from neuroimaging and treated as non-mutually exclusive. Laboratory variables obtained at admission included triglycerides, total cholesterol, high-density lipoprotein cholesterol, low-density lipoprotein cholesterol, fasting glucose, glycated hemoglobin (HbA1c), homocysteine, uric acid, creatinine, blood urea nitrogen (BUN), C-reactive protein (CRP), prothrombin time, and fibrinogen.

Alcohol intake was assessed as the average number of standard drinks consumed per day over the preceding 12 months, based on self-report. Alcohol consumption was converted to grams of ethanol per day (1 standard drink = 10 g ethanol) and modeled as a continuous variable. For descriptive comparisons, drinking amount was calculated among current and former drinkers only; for regression analyses, non-drinkers were coded as 0 g ethanol/day.

### 2.4 Cognitive assessment

Cognitive function was evaluated using the Chinese version of the Montreal Cognitive Assessment (MoCA), administered in Chinese by trained neurologists. The MoCA assesses seven cognitive domains (visuospatial/executive function, naming, attention, language, abstraction, delayed recall, and orientation), with a total score ranging from 0 to 30. Cognitive impairment was defined as a MoCA score <26, with a 1-point education adjustment applied for individuals with ≤12 years of formal education. This adjustment was applied prior to group assignment.

### 2.5 Frailty assessment

Frailty was assessed using the Edmonton Frail Scale (EFS), which yields a total score ranging from 0 to 17. Frailty categories were defined as non-frail (0–5), pre-frail (6–7), and frail (≥ 8). For item-level analyses, pre-frail and frail categories were combined. Because the EFS includes a cognition-related item, potential conceptual overlap with MoCA-based cognitive assessment was acknowledged, and a sensitivity analysis was performed excluding this item (EFS total minus cognition item).

All cognitive and frailty assessments were performed independently by three trained neurologists who were blinded to participants’ clinical and laboratory data.

### 2.6 Statistical analysis

Statistical analyses were conducted using SPSS version 26.0 (IBM Corp., Armonk, NY, USA) and R version 4.3.1 (R Foundation for Statistical Computing, Vienna, Austria). Continuous variables are presented as mean ± standard deviation or median with interquartile range, as appropriate, and categorical variables as counts and percentages. Group comparisons were performed using independent-samples t tests or Mann–Whitney *U* tests for continuous variables and chi-square tests or Fisher’s exact tests for categorical variables. Baseline and item-level *p*-values were considered descriptive and were not adjusted for multiple comparisons.

Associations between EFS total score and MoCA domain scores were examined using Spearman rank correlation coefficients (*ρ*), with sample size reported for each analysis. Multivariable logistic regression was used to identify independent predictors of cognitive impairment. Candidate variables were selected based on clinical relevance and univariable screening, with NIHSS score forced into the model. Education level was coded as an ordinal variable (1 = primary or less, 2 = middle/high school, 3 = college or above). Multicollinearity was assessed using variance inflation factors.

The multivariable model was fitted using complete-case analysis. Odds ratios (ORs) with 95% confidence intervals (CIs) are reported. Model discrimination was assessed using the area under the receiver operating characteristic curve (AUC), and calibration was evaluated using the Brier score with apparent calibration assessed on the development dataset (optimism not corrected). A two-sided *p*-value <0.05 was considered statistically significant.

A sensitivity analysis was conducted by recalculating the EFS total score excluding the cognition item and repeating the correlation and multivariable regression analyses.

## 3 Results

### 3.1 Patient characteristics

A total of 179 older adults (≥65 years) hospitalized with acute cerebral infarction were included. Using the education-adjusted MoCA definition (MoCA <26 with a 1-point adjustment for ≤12 years of education applied prior to group assignment), 117 patients (65.4%) were classified as cognitively impaired (CI) and 62 (34.6%) as cognitively unimpaired (CU), consistent with the prespecified grouping strategy.

### 3.2 Baseline demographic, clinical, and laboratory characteristics

Baseline characteristics by cognitive status are summarized in Table 1. Compared with CU patients, CI patients were older (73.03 ± 5.95 vs. 71.27 ± 4.18 years; *p* = 0.023) and had higher stroke severity on admission (NIHSS: 3.85 ± 2.80 vs. 2.77 ± 2.61; *p* = 0.011). Education level differed between groups (*p* = 0.001), with a higher proportion of CI patients having primary education or less (31.6% vs. 8.1%) and a lower proportion having college education or above (16.2% vs. 30.6%).

**Table 1:** Baseline characteristics.

Variable	CU (*n* = 62)	CI (*n* = 117)	Statistic	*p*-value
Females sex (%)	25 (40.3)	42 (35.9)	0.339	0.561
Age (yr)	71.27 ± 4.18	73.03 ± 5.95	−2.301	0.023
BMI (kg/m^2^)	24.76 ± 2.88	25.18 ± 3.69	−0.814	0.417
Smoking duration (years)	36.90 ± 13.55	37.13 ± 14.04	−0.063	0.950
Cigarettes per day	15.67 ± 9.07	17.09 ± 10.63	−0.561	0.578
Smoking status (%)			1.100	0.577
Never	38 (61.3)	67 (57.3)		
Current	18 (29.0)	42 (35.9)		
Former	6 (9.7)	8 (6.8)		
Drinking duration (years)	34.94 ± 14.05	37.34 ± 15.42	−0.578	0.567
Daily alcohol intake (g ethanol/day)	17.22 ± 6.69	31.32 ± 21.95	−3.618	0.001
Drinking status (%)			0.236	0.889
Never	43 (69.4)	77 (65.8)		
Current	16 (25.8)	34 (29.1)		
Former	3 (4.8)	6 (5.1)		
Education level (%)			14.162	0.001
Primary or less	5 (8.1)	37 (31.6)		
Middle/high school	38 (61.3)	61 (52.1)		
College or above	19 (30.6)	19 (16.2)		
Diabetes (%)	19 (30.6)	42 (35.9)	0.498	0.481
Hypertension (%)	43 (69.4)	73 (62.4)	0.861	0.353
Atrial fibrillation/flutter (%)	2 (3.2)	5 (4.3)	0.118	0.731
Stable angina (%)	1 (1.6)	3 (2.6)	0.168	0.682
Unstable angina (%)	1 (1.6)	2 (1.7)	0.002	0.962
Myocardial infarction (%)	4 (6.5)	15 (12.8)	1.733	0.188
NIHSS score	2.77 ± 2.61	3.85 ± 2.80	−2.567	0.011
Infarction site (%)				
Cerebellum	1 (1.6)	3 (2.6)	0.168	0.682
Frontal	7 (11.3)	16 (13.7)	0.206	0.650
Parietal	6 (9.7)	14 (12.0)	0.214	0.644
Temporal	0 (0.0)	4 (3.4)	2.168	0.141
Occipital	0 (0.0)	5 (4.3)	2.726	0.099
Limbic	21 (33.9)	40 (34.2)	0.002	0.966
Internal capsule	3 (4.8)	7 (6.0)	0.101	0.751
Basal ganglia	42 (67.7)	62 (53.0)	3.622	0.057
Midbrain	1 (1.6)	5 (4.3)	0.886	0.347
Diencephalon	5 (8.1)	22 (18.8)	3.649	0.056
Pons	13 (21.0)	19 (16.2)	0.617	0.432
Triglycerides (mmol/L)	1.94 ± 1.43	1.71 ± 1.13	1.111	0.269
Total cholesterol (mmol/L)	4.52 ± 1.12	4.53 ± 1.08	−0.045	0.964
HDL (mmol/L)	1.15 ± 0.31	1.20 ± 0.36	−0.870	0.386
LDL (mmol/L)	2.71 ± 0.95	2.77 ± 1.01	−0.436	0.663
Fasting glucose (mmol/L)	7.21 ± 3.19	7.41 ± 3.24	−0.384	0.701
HbA1c (%)	6.78 ± 1.76	7.01 ± 1.96	−0.780	0.437
Homocysteine (umol/L)	13.84 ± 4.39	15.93 ± 11.38	−1.491	0.139
Uric acid (umol/L)	315.13 ± 91.05	322.05 ± 79.69	−0.496	0.621
Creatinine (umol/L)	65.98 ± 14.23	74.13 ± 46.96	−1.706	0.090
BUN (mmol/L)	5.14 ± 1.42	5.69 ± 1.92	−2.135	0.034
CRP (mg/L)	6.11 ± 14.80	5.66 ± 11.71	0.143	0.887
Prothrombin time (s)	13.72 ± 4.65	13.62 ± 3.21	0.155	0.877
Fibrinogen (g/L)	291.98 ± 74.90	284.82 ± 83.49	0.578	0.564

Smoking/drinking duration and daily alcohol intake are calculated among current/former smokers or drinkers only.

Infarction sites are non-mutually exclusive; *p*-values are per site (no overall site test).

Baseline *p*-values are descriptive and not adjusted for multiple testing.

Statistic denotes independent-samples *t*-test or Mann–Whitney *U* test for continuous variables and chi-square test or Fisher’s exact test for categorical variables.

Alcohol exposure also differed between groups. Among current/former drinkers, CI patients reported higher daily ethanol intake (31.32 ± 21.95 vs. 17.22 ± 6.69 g ethanol/day; *p* = 0.001), whereas drinking status distribution (never/current/former) did not differ significantly (*p* = 0.889). Other vascular comorbidities (e.g., diabetes, hypertension, atrial fibrillation/flutter, myocardial infarction) were comparable between groups (all *p* > 0.05).

For laboratory indices measured at admission, blood urea nitrogen (BUN) was higher in CI than CU patients (5.69 ± 1.92 vs. 5.14 ± 1.42 mmol/L; *p* = 0.034), while other markers including lipid profile, glucose/HbA1c, homocysteine, uric acid, creatinine, CRP, prothrombin time, and fibrinogen showed no significant between-group differences (all *p* > 0.05). Infarction sites were analyzed as non-mutually exclusive categories; site-specific comparisons are reported in Table 1, and no significant differences were observed between groups for any specific site. Baseline *p*-values are descriptive and were not adjusted for multiple testing.

### 3.3 Frailty status and EFS item-level comparisons

Frailty findings are presented in Table 2. Based on EFS total score categories (non-frail 0–5; pre-frail 6–7; frail ≥ 8), the overall prevalence of pre-frailty/frailty (combined) was 45.8% (82/179). The proportion classified as pre-frail/frail was higher in the CI group than in the CU group (59.8% [70/117] vs. 19.4% [12/62]; chi-square = 26.742; *p* < 0.001).

**Table 2:** EFS item-level comparisons.

Item	CU (*n* = 62)	CI (*n* = 117)	Chi-square	*p*-value
Frailty category			26.742	<0.001
Non-frail	50 (80.6)	47 (40.2)		
Pre-frail/frail	12 (19.4)	70 (59.8)		
Clock-drawing test			30.195	<0.001
No error	49 (79.0)	43 (36.8)		
Spacing errors	9 (14.5)	35 (29.9)		
Other errors	4 (6.5)	39 (33.3)		
Hospitalizations in past year			3.673	0.159
0	44 (71.0)	66 (56.4)		
1–2	14 (22.6)	41 (35.0)		
>2	4 (6.5)	10 (8.5)		
Self-rated health			7.406	0.025
Good	29 (46.8)	33 (28.2)		
Fair	22 (35.5)	46 (39.3)		
Poor	11 (17.7)	38 (32.5)		
Dependence in IADL			16.461	<0.001
0–1 tasks	53 (85.5)	65 (55.6)		
2–4 tasks	5 (8.1)	22 (18.8)		
5–8 tasks	4 (6.5)	30 (25.6)		
Social support availability			3.467	0.177
Always	55 (88.7)	93 (79.5)		
Sometimes	4 (6.5)	19 (16.2)		
Rarely	3 (4.8)	5 (4.3)		
≥5 medications long-term	20 (32.3)	41 (35.0)	0.140	0.708
Often forget medications	5 (8.1)	17 (14.5)	1.571	0.210
Recent weight loss	11 (17.7)	39 (33.3)	4.894	0.027
Depressed mood	15 (24.2)	50 (42.7)	6.024	0.014
Urinary incontinence	7 (11.3)	35 (29.9)	7.827	0.005
Unable to perform heavy housework	22 (35.5)	71 (60.7)	10.310	0.001
Unable to climb 2 flights of stairs	15 (24.2)	54 (46.2)	8.250	0.004
Unable to walk 1,000 meters	14 (22.6)	47 (40.2)	5.582	0.018

Frailty category based on EFS total score: non-frail = 0–5, pre-frail = 6–7, frail ≥8; pre-frail and frail were merged for analysis.

*p*-values are descriptive and not adjusted for multiple testing.

Item-level comparisons (exploratory; unadjusted for multiple testing) showed that CI patients more frequently exhibited clock-drawing errors (*p* < 0.001), poorer self-rated health (*p* = 0.025), greater IADL dependence (*p* < 0.001), recent weight loss (*p* = 0.027), depressed mood (*p* = 0.014), urinary incontinence (*p* = 0.005), and limitations in physical function (heavy housework, climbing stairs, and walking 1,000 meters; all *p* < = 0.018). In contrast, hospitalizations in the past year, social support availability, polypharmacy (≥5 long-term medications), and medication nonadherence were not significantly different between groups (all *p* > 0.05).

### 3.4 Correlation between frailty and cognitive domains

As shown in Table 3, EFS total score was inversely correlated with global cognition and all raw MoCA domain scores using Spearman rank correlations (*n* = 179 for each domain). The strongest associations were observed for total MoCA score (*ρ* = −0.572; *p* < 0.001) and visuospatial/executive function (*ρ* = −0.556; *p* < 0.001), followed by orientation (*ρ* = −0.479; *p* < 0.001) and attention (*ρ* = −0.417; *p* < 0.001). Correlations for the remaining domains were also significant (all *p* < = 0.008).

**Table 3:** Spearman correlations between EFS total score and MoCA domains.

MoCA domain	*ρ*	*p*-value
Visuospatial/executive	−0.556	<0.001
Naming	−0.197	0.008
Attention	−0.417	<0.001
Language	−0.308	<0.001
Abstraction	−0.390	<0.001
Delayed recall	−0.373	<0.001
Orientation	−0.479	<0.001
Total MoCA score	−0.572	<0.001

Spearman rank correlation, *n* = 179 for each domain.

### 3.5 Multivariable predictors of cognitive impairment

Multivariable logistic regression results are shown in Table 4. The final model was fitted using complete-case analysis (*n* = 173). After adjustment, higher daily alcohol intake (per 10 g ethanol/day; OR 1.43, 95% CI 1.07–1.90; *p* = 0.016) and higher frailty score (per 1-point increase; OR 1.34, 95% CI 1.17–1.55; *p* < 0.001) were independently associated with CI, while higher education level (ordinal; OR 0.59, 95% CI 0.37–0.94; *p* = 0.027) was associated with lower odds of CI. Age, NIHSS score, and BUN did not reach statistical significance in the adjusted model (all *p* ≥ 0.096).

**Table 4:** Multivariable logistic regression for cognitive impairment.

Variable	*β*	SE	OR (95% CI)	*p*-value
Intercept	−3.001	2.874	0.05 (0.00–13.90)	0.296
Age	0.023	0.039	1.02 (0.95–1.11)	0.556
Daily alcohol intake (per 10 g ethanol/day)	0.355	0.147	1.43 (1.07–1.90)	0.016
Education level	−0.524	0.238	0.59 (0.37–0.94)	0.027
BUN	0.197	0.118	1.22 (0.97–1.54)	0.096
NIHSS score	0.094	0.081	1.10 (0.94–1.29)	0.248
Frailty score	0.296	0.071	1.34 (1.17–1.55)	<0.001

Model fit: AUC = 0.821, Brier score = 0.163.

Education level was coded as an ordinal variable: 1 = primary or less, 2 = middle/high school, 3 = college or above.

Model performance indicated good discrimination (AUC = 0.821) and adequate calibration (Brier score = 0.163) on the development dataset.

### 3.6 Sensitivity analysis (EFS total minus cognition item)

After removing the EFS cognition item (clock-drawing) from the total score, the association between frailty and cognition remained robust. In the multivariable model (*n* = 173; CI = 112, CU = 61), the revised frailty score remained independently associated with CI (OR 1.31, 95% CI 1.13–1.51; *p* < 0.001). Alcohol intake (per 10 g ethanol/day) and education level remained significant, while NIHSS and BUN remained non-significant (Table 5).

**Table 5:** Sensitivity analysis (EFS total minus cognition item.).

Variable	*β*	SE	OR (95% CI)	*p*-value
Age	0.030	0.038	1.03 (0.96–1.11)	0.427
Daily alcohol intake (per 10 g ethanol/day)	0.364	0.143	1.44 (1.09–1.91)	0.011
Education level	−0.567	0.237	0.57 (0.36–0.90)	0.017
BUN	0.203	0.117	1.23 (0.97–1.54)	0.082
NIHSS score	0.107	0.080	1.11 (0.95–1.30)	0.179
Frailty score (minus cognition)	0.269	0.074	1.31 (1.13–1.51)	<0.001

Spearman correlations using the revised frailty score also remained significant across all MoCA domains, with attenuated but consistent effect sizes (total MoCA *ρ* = −0.485; *p* < 0.001).

## 4 Discussion

In this hospital-based cross-sectional study of older adults hospitalized with acute cerebral infarction, cognitive impairment defined by an education-adjusted MoCA threshold was common. Several clinical features distinguished CI from CU patients, including older age, greater stroke severity at admission, lower educational attainment, higher daily ethanol intake among current/former drinkers, and a higher burden of frailty. Frailty was consistently related to cognition across complementary analyses: CI patients were more likely to be pre-frail/frail, EFS item-level deficits clustered in functional/psychological domains, and EFS total score correlated negatively with global MoCA and all cognitive domains. In multivariable modeling, higher frailty score and higher alcohol exposure (scaled per 10 g ethanol/day) were independently associated with CI, while higher education was protective, supporting the clinical relevance of frailty and modifiable lifestyle factors in post-stroke cognitive vulnerability.

Frailty and cognitive performance are closely linked in older populations, with frailty showing inverse associations with global cognition and multiple cognitive domains (Lundberg et al., 2024). In stroke populations, frailty has been recognized as a marker of reduced physiological reserve and worse neurocognitive outcomes, although effect sizes and time horizons vary across studies and outcome definitions (Searle and Rockwood, 2015).

Mechanistically, frailty may capture cumulative multisystem decline (mobility limitation, nutritional deficits, mood symptoms, functional dependence, and social vulnerability) that constrains cognitive reserve and recovery after cerebrovascular injury (Evans et al., 2022). The relationship may be bidirectional: frailty may lower neural resilience to ischemic injury, while cognitive impairment could restrict independence in instrumental activities of daily living (IADL), thereby accelerating physical decline (Fu et al., 2018). Temporality cannot be established due to the cross-sectional design. In our data, the CI group showed higher frequencies of impairments in IADL dependence, self-rated health, depressed mood, weight loss, urinary incontinence, and physical function items, suggesting that cognitive impairment co-occurs with broader geriatric syndromes rather than presenting in isolation. Because these associations were observed without significant differences in CRP, we do not interpret the findings as evidence of systemic inflammation in this cohort. Instead, the results highlight functional and psychosocial aspects of frailty, such as depression and social isolation, as key correlates of cognition in the early post-stroke period (Bai et al., 2024).

The Edmonton Frail Scale offers practical advantages in acute care because it is brief and multidimensional, and its reliability and validity have been established in older adults (Lee et al., 2025). The strong correlations between EFS total score and MoCA global score (and the visuospatial/executive and attention domains in particular) suggest that frailty screening may help identify patients who warrant earlier and more comprehensive cognitive evaluation and tailored rehabilitation strategies (Vahedi et al., 2022). This specific impairment in executive and attention functions is consistent with literature linking frailty to cerebral small vessel disease, which preferentially disrupts frontal-subcortical circuits (Chang et al., 2022).

A key methodological refinement was the use of clinically meaningful alcohol units. We quantified alcohol intake in grams of ethanol per day and modeled the association per 10 g ethanol/day (equivalent to one standard drink), yielding an adjusted OR of 1.43 for cognitive impairment per 10 g/day increase. This scaling improves interpretability and allows contextual comparison with prior work that reports risk in grams/day or drinks/day. Existing evidence on alcohol and cognition is mixed and often nonlinear, with several studies and meta-analyses reporting U- or J-shaped dose–response patterns depending on cognitive endpoints, age, sex, and confounding structure (Sabia et al., 2018). In contrast, our cross-sectional data within an acute stroke cohort support a monotonic association between higher ethanol intake and higher odds of cognitive impairment, even after accounting for education and frailty. This aligns with findings suggesting that the neurotoxic and vascular effects of increasing alcohol volume may outweigh potential benefits in vulnerable populations, supporting a dose-dependent risk (Sun et al., 2024).

Several factors may explain differences between our results and U−/J-shaped patterns described in community cohorts. First, stroke patients constitute a clinically selected population in whom alcohol may be more strongly linked to cerebrovascular burden and cognitive vulnerability. Second, cross-sectional estimates are susceptible to residual confounding and sick quitter effects (e.g., former drinkers with comorbidities grouped with abstainers), which can distort apparent protective associations in observational data. Third, our descriptive comparisons of alcohol dose were restricted to current/former drinkers, whereas regression analyses treated non-drinkers as 0 g/day; differences in operationalization and cohort composition can influence the shape of observed associations. Accordingly, while our findings support alcohol exposure as an independent correlate of cognitive impairment in this setting, causal inference is not warranted and prospective data with repeated cognitive assessments are needed to define trajectory and potential thresholds.

Education remained independently protective against cognitive impairment in multivariable analysis, consistent with the concept of cognitive reserve and the known influence of educational attainment on screening performance and post-stroke cognitive resilience (Elkana et al., 2025; Sumowski et al., 2013). The education adjustment and grouping strategy were implemented according to established MoCA guidance, which recommends a 1-point correction for individuals with ≤12 years of education. Although stroke severity (NIHSS) and BUN differed between groups in baseline comparisons, neither was statistically significant after multivariable adjustment. This pattern suggests that the observed unadjusted associations may reflect shared variance with frailty, education, and alcohol exposure, or limited power for smaller independent effects in the adjusted model.

The sensitivity analysis excluding the EFS cognition item showed that the frailty-cognition association remained significant and directionally consistent in both correlations and multivariable regression. This supports the robustness of the primary findings and reduces concern that item-level overlap between EFS and MoCA accounts for the observed associations.

Several limitations merit emphasis. First, the cross-sectional design precludes causal inference and does not permit determination of temporal relationships among alcohol intake, frailty, and cognitive impairment. Second, assessments were conducted 7–14 days after admission to allow stabilization; this timing may have introduced selection bias by excluding patients with the most severe strokes or those unable to complete testing, potentially underestimating the overall cognitive impairment burden and influencing observed associations. Third, item-level overlap between the EFS and MoCA cognitive items may have inflated the observed associations between frailty and cognition. Fourth, the multivariable model used complete-case analysis, which may introduce bias if missingness is not completely at random. Finally, this was a single-center study, and external validation in other settings is required.

### 4.1 Conclusion

In this cross-sectional study of older patients hospitalized with acute cerebral infarction, cognitive impairment defined by education-adjusted MoCA criteria showed an association with higher frailty burden and higher daily ethanol intake (per 10 g/day), whereas higher educational attainment was associated with lower odds of cognitive impairment. These findings show an association between cognitive impairment and higher frailty burden and daily ethanol intake (per 10 g/day), while higher educational attainment was associated with lower odds of cognitive impairment. These findings support the clinical value of integrating brief frailty screening and standardized alcohol exposure assessment into early in-hospital evaluation, while emphasizing that longitudinal studies are needed to clarify temporality and causal pathways.

## Data Availability

The original contributions presented in the study are included in the article/supplementary material, further inquiries can be directed to the corresponding author.

## References

[ref1] Bai Y. Chen Y. Tian M. Gao J. Song Y. Zhang X. . (2024). The relationship between social isolation and cognitive frailty among community-dwelling older adults: the mediating role of depressive symptoms. Clin. Interv. Aging 19, 1079–1089. doi: 10.2147/CIA.S461288, 38911673 PMC11192202

[ref2] Brainin M. Tuomilehto J. Heiss W. D. Bornstein N. M. Bath P. M. Teuschl Y. . (2015). Post-stroke cognitive decline: an update and perspectives for clinical research. Eur. J. Neurol. 22, 229–e16. doi: 10.1111/ene.12626 25492161

[ref3] Burton J. K. Stewart J. Blair M. Oxley S. Wass A. Taylor-Rowan M. . (2022). Prevalence and implications of frailty in acute stroke: systematic review & meta-analysis. Age Ageing 51:afac064. doi: 10.1093/ageing/afac064, 35352795 PMC9037368

[ref4] Chang Y. S. Wang C. J. Wu C. H. Wu Y. H. Lee H. N. (2022). Frailty is associated with frontal cortex-related cognitive function in patients with Alzheimer disease. J. Geriatr. Psychiatry Neurol. 35, 544–549. 33977812 10.1177/08919887211016062

[ref12] Costa Novo J. Rieffel E. Velarde G. C. Costa F. Barros P. Veloso M. . (2024). Shorter reperfusion time in stroke is associated with better cognition. Can. J. Neurol. Sci. 51, 644–649. doi: 10.1017/cjn.2023.321 38052728

[ref5] Elkana O. Beheshti I. Alzheimer’s Disease Neuroimaging Initiative (2025). The protective role of education in white matter lesions and cognitive decline. Brain Cogn. 187:106304. doi: 10.1016/j.bandc.2025.106304 40288270

[ref6] Evans N. R. Todd O. M. Minhas J. S. Fearon P. Harston G. W. Mant J. . (2022). Frailty and cerebrovascular disease: concepts and clinical implications for stroke medicine. Int. J. Stroke 17, 251–259. doi: 10.1177/17474930211034331, 34282986 PMC8864332

[ref7] Fu C. Li Z. Mao Z. (2018). Association between social activities and cognitive function among the elderly in China: a cross-sectional study. Int. J. Environ. Res. Public Health 15:231. doi: 10.3390/ijerph15020231, 29385773 PMC5858300

[ref8] Guo X. Pei J. Ma Y. Cui Y. Guo J. Wei Y. . (2023). Cognitive frailty as a predictor of future falls in older adults: a systematic review and meta-analysis. J. Am. Med. Dir. Assoc. 24, 38–47. doi: 10.1016/j.jamda.2022.10.011, 36423679

[ref9] Lee Z. R. Cheng L. J. Yeo Y. Y. Rolfson D. Wu X. V. (2025). Measurement properties of the Edmonton frail scale in older adults: a systematic review and meta-analysis. Int. J. Nurs. Stud. 170:105161. doi: 10.1016/j.ijnurstu.2025.105161, 40706437

[ref10] Lundberg K. Elmståhl S. Wranker L. S. Ekström H. (2024). The association between physical frailty and cognitive performance in older adults aged 60 to 96 years: data from the “good aging in Skåne” (GÅS) Swedish population study. Ann. Geriatr. Med. Res. 28, 330–341. doi: 10.4235/agmr.24.0055, 38782711 PMC11467518

[ref11] Luo Y. Su B. Zheng X. (2021). Trends and challenges for population and health during population aging-China, 2015–2050. China CDC Wkly 3, 593–598. doi: 10.46234/ccdcw2021.158, 34594944 PMC8393078

[ref13] Sabia S. Fayosse A. Dumurgier J. Dugravot A. Akbaraly T. Britton A. . (2018). Alcohol consumption and risk of dementia: 23 year follow-up of Whitehall II cohort study. BMJ 362:k2927. doi: 10.1136/bmj.k2927, 30068508 PMC6066998

[ref14] Searle S. D. Rockwood K. (2015). Frailty and the risk of cognitive impairment. Alzheimer's Res. Ther. 7:54. doi: 10.1186/s13195-015-0140-3, 26240611 PMC4523015

[ref15] Singh M. Stewart R. White H. (2014). Importance of frailty in patients with cardiovascular disease. Eur. Heart J. 35, 1726–1731. doi: 10.1093/eurheartj/ehu197, 24864078 PMC4565652

[ref16] Sumowski J. F. Chiaravalloti N. Krch D. Paxton J. DeLuca J. (2013). Education attenuates the negative impact of traumatic brain injury on cognitive status. Arch. Phys. Med. Rehabil. 94, 2562–2564. doi: 10.1016/j.apmr.2013.07.023, 23932968

[ref17] Sun M. Zheng Q. Wang L. Wang R. Cui H. Zhang X. . (2024). Alcohol consumption during adolescence alters the cognitive function in adult male mice by persistently increasing levels of DUSP6. Mol. Neurobiol. 61, 3161–3178. doi: 10.1007/s12035-023-03794-x, 37978157

[ref18] Taylor-Rowan M. Hafdi M. Drozdowska B. Elliott E. Wardlaw J. Quinn T. J. . (2023). Physical and brain frailty in ischaemic stroke or TIA: shared occurrence and outcomes. A cohort study. Eur. Stroke J. 8, 1011–1020. doi: 10.1177/23969873231186480 37421136 PMC10683729

[ref19] Taylor-Rowan M. Keir R. Cuthbertson G. Shaw R. Drozdowska B. Elliott E. . (2019). Pre-stroke frailty is independently associated with post-stroke cognition: a cross-sectional study. J. Int. Neuropsychol. Soc. 25, 501–506. doi: 10.1017/S1355617719000092, 30821222

[ref20] Vahedi A. Eriksdotter M. Ihle-Hansen H. Wyller T. B. Øksengård A. R. Fure B. . (2022). Cognitive impairment in people with physical frailty using the phenotype model: a systematic review and meta analysis. Int. J. Geriatr. Psychiatry 37:10.1002/gps.5822. doi: 10.1002/gps.5822 PMC9828066 36221235

[ref21] Xu Z. Weng X. Cao L. Liang D. Zeng F. Chen S. . (2024). Correlation analysis of serum 3-NT, NPASDP-4, and S100β protein levels with cognitive function in patients diagnosed with cerebral infarction. Altern. Ther. Health Med. 30, 54–59. 37944966

